# Benin responds to covid-19: sanitary cordon without generalized containment or lockdown?

**DOI:** 10.1186/s41182-020-00235-6

**Published:** 2020-06-15

**Authors:** Issideen Ayinla Osseni

**Affiliations:** 1 Department of Clinical Medicine, International School, Jinan University, 510632 Guangzhou, People’s Republic of China; 2 Department of Medicine, UAC Health Sciences Faculty, Cotonou, Benin

**Keywords:** COVID-19, Low-income country, Containment, Lockdown, Sanitary cordon, Benin

## Abstract

Since the novel coronavirus disease 2019 (COVID-19) has been identified in Wuhan, China, in the last week of December, the virus has spread across nations and continents affecting over 3 million people and putting the whole world to a halt. Nations across the globe went on lockdown in an attempt to contain the spread of the virus and curb its propagation curve. Affected African countries did the same except for Benin, which opted for a sanitary cordon around the affected regions with free movement of people. The biggest challenge is the effectiveness of this measure.

To the Editor,

In a matter of 4 months, COVID-19 has spread across nations and is currently affecting all continents except for Antarctica. On 11 March 2020, it was declared a pandemic by the World Health Organization [1]. The African Continent has the second least number of cases and deaths from COVID-19 after Oceania [2]. In an attempt to flatten the curve of the virus propagation, nations across the globe opted for generalized containment or lockdown measures causing malls, bars, restaurants, shops, etc. to close [3, 4] allowing African countries to follow suit. Though enforcing this mitigation measure will negatively impact the economy, it will subsequently cause a decline in the annual GDP [5]. However, the economic repercussions will differ depending on the living conditions and access to essential services of a country’s population. Low-income countries like Benin will face tremendous economic hardship because there is a high reliance on informal activities [6]. Since a large portion of Benin’s population depends on a daily or weekly wage, the economic drawback of such mitigation measures undoubtedly outweighs the benefit of enforcing a generalized containment or lockdown [7, 8].

In an attempt to contain COVID-19, in early February, Benin government prevention efforts were to screen all flight passengers coming into the country. The Ministry of Health installed a temperature scanner, handwashing apparatus, and an isolation room in the country’s international airport as air traffic is a major facilitator in COVID-19 distribution [7, 8]. As COVID-19 first report makes the news in Benin’s capital, Porto-Novo on 16 March 2020, the government introduced stricter measures to prevent the rise in cases (Table 1).

**Table 1:** Health protection measures against COVID-19 in Benin, 11 February–27 April 2020

Date	Public health measure implemented	Place	Authority
11 February	-Health checkpoint for passengers at airport and land borders -Self isolation of passengers that have been to affected countries	Airport, borders	Ministry of Health
17 February	-Entry ban put on passengers that have been to China in the past 14 days at the airport and land borders	Airport	Government
03 March	Toll free phone numbers are made available to alert on suspicious cases or people that have arrived from China bypassing the airport or land borders and people that have not self-isolate	Nationwide	Ministry of Health
18 March	-Limit entry and exit at land borders to goods only -Restrict entry visa to the country -Mandatory supervised quarantine for 14 for people coming in -Suspension of all public events and unnecessary shops, while restaurants, supermarkets, schools and universities remained open -Stocks of masks are made available to pharmacies, supermarkets	Nationwide	Government
23 March	-Early Easter holidays so that schools and universities can close -Sanitary cordon starting from 30th of March around 12 towns or cities; movement of people and goods within the sanitary cordon is permitted -Movement of people outside the cordon to the sanitary cordon is prohibited (vice versa), while the transport of goods between the two zones is authorized	Nationwide/affected towns or cities	Government
	Table 1 (continued)		
07 April	-Mandatory wear of mask inside the sanitary cordon starting from 08 April	Affected towns or cities	Government
10 April	-Schools, universities, nurseries, places of worship would remain closed till 10 May while essential shops remained open	Nationwide	Government
14 April	Three towns or cities are added to the sanitary cordon (Fig. 2)	Affected towns or cities	Government
27 April	-Mandatory wear of mask nationwide starting from 28 April	Nationwide	Government

In his address to the country on national television on 29 March 2020, Benin’s President, Patrice Talon explained how alarming the situation was, he called on the public responsibility and tried to explain the different mitigation measures his government is putting in place [10]. He recognized how the majority of the population rely on informal labor and clarified to why it was virtually impossible to take harsher containment measures and locking down the country. Furthermore, he explained how a sanitary cordon is more appropriate to the country’s reality and which areas are to be included (Fig. 1) since failure to quickly contain the virus would result in its significant spread throughout the rest of the country [11]. Besides, he insisted that people inside the sanitary cordon have freedom of movement while respecting handwashing and hygiene measures.

**Fig. 1:**
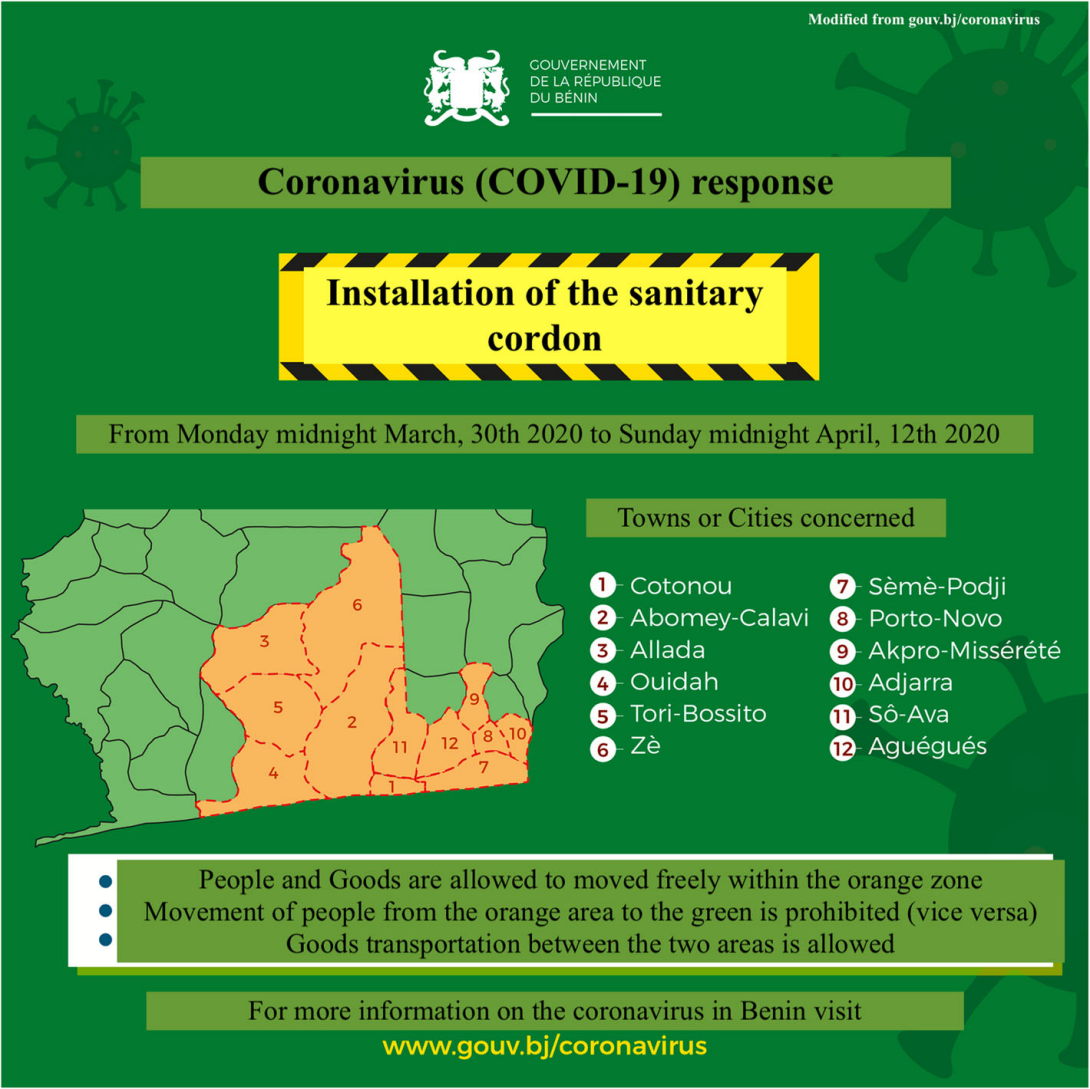
Sanitary cordon as of March 30 around 12 towns or cities. Data derived from “Gouvernement de la Republique du Benin” COVID-19 webpage (https://www.gouv.bj/actualites/categorie/coronavirus%2D%2Dcovid-19-/) [9]

Mitigation measures at first had been promoting handwashing and hygiene by installing handwashing equipment on the streets, along with social distancing. Individuals that were in Chinese territories during the past 14 days were not allowed in Benin and those coming from other affected countries were asked to self-isolate. Afterward, the government added on stricter measures such as the closure of churches, mosques, clubs, bars while restaurants and supermarkets were left open [9] Fig. 2.

**Fig. 2:**
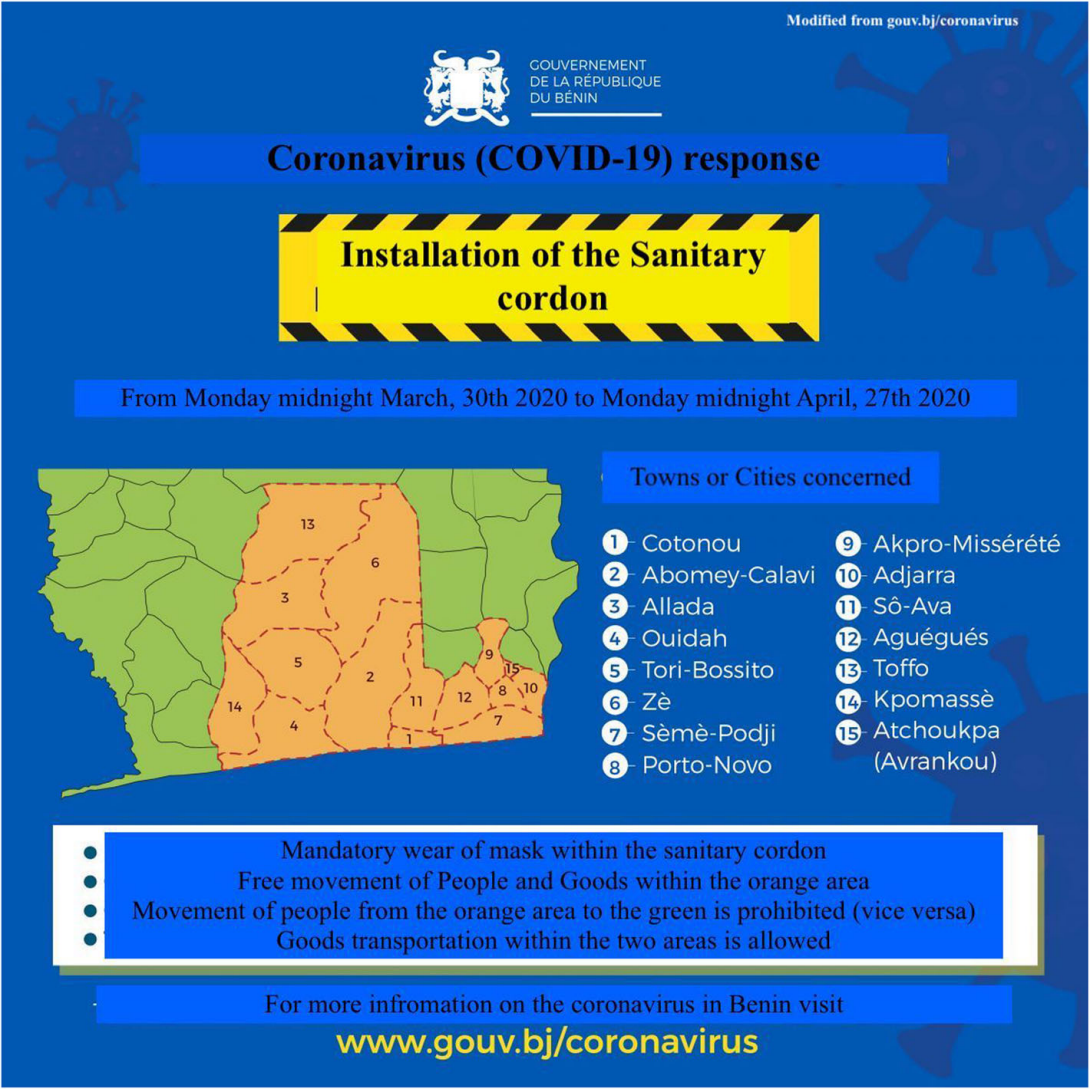
Sanitary Cordon as of April, 14 around 15 towns or cities. Data derived from “Gouvernement de la Republique du Benin” COVID-19 webpage (https://www.gouv.bj/actualites/categorie/coronavirus%2D%2Dcovid-19-/) [9]

Although the country is already burdened by infectious diseases such as Lassa fever, malaria, and tuberculosis, adding COVID-19 pandemic, these mitigation measures seem inappropriate and insufficient for a low-income country. Additionally, the country faces several health challenges such as limited access to beds, ventilators, intensive care units, laboratories, and is profoundly short on specially trained clinicians [12, 13]. Hitherto, Benin’s government was able to contain the virus to three community cases while the rest of the cases were diagnosed and restrained during the mandatory 14 days supervised quarantine. As of 28 April 2020, the country counts 64 COVID-19 cases (Fig. 3), 30 cases under treatment, 33 cured cases, and one death [9]. An element of luck was that, after the Lassa fever outbreak in January 2016, with the support of the World Health Organization (WHO), there were significant improvements in the surveillance, emergency response, and prevention of infectious diseases. The capacities of Benin with its neighboring countries were boosted [14]. Today, the government makes use of social media to pass information to the population in different languages and dialects to combat misinformation and safeguard the national public health and safety

**Fig. 3:**
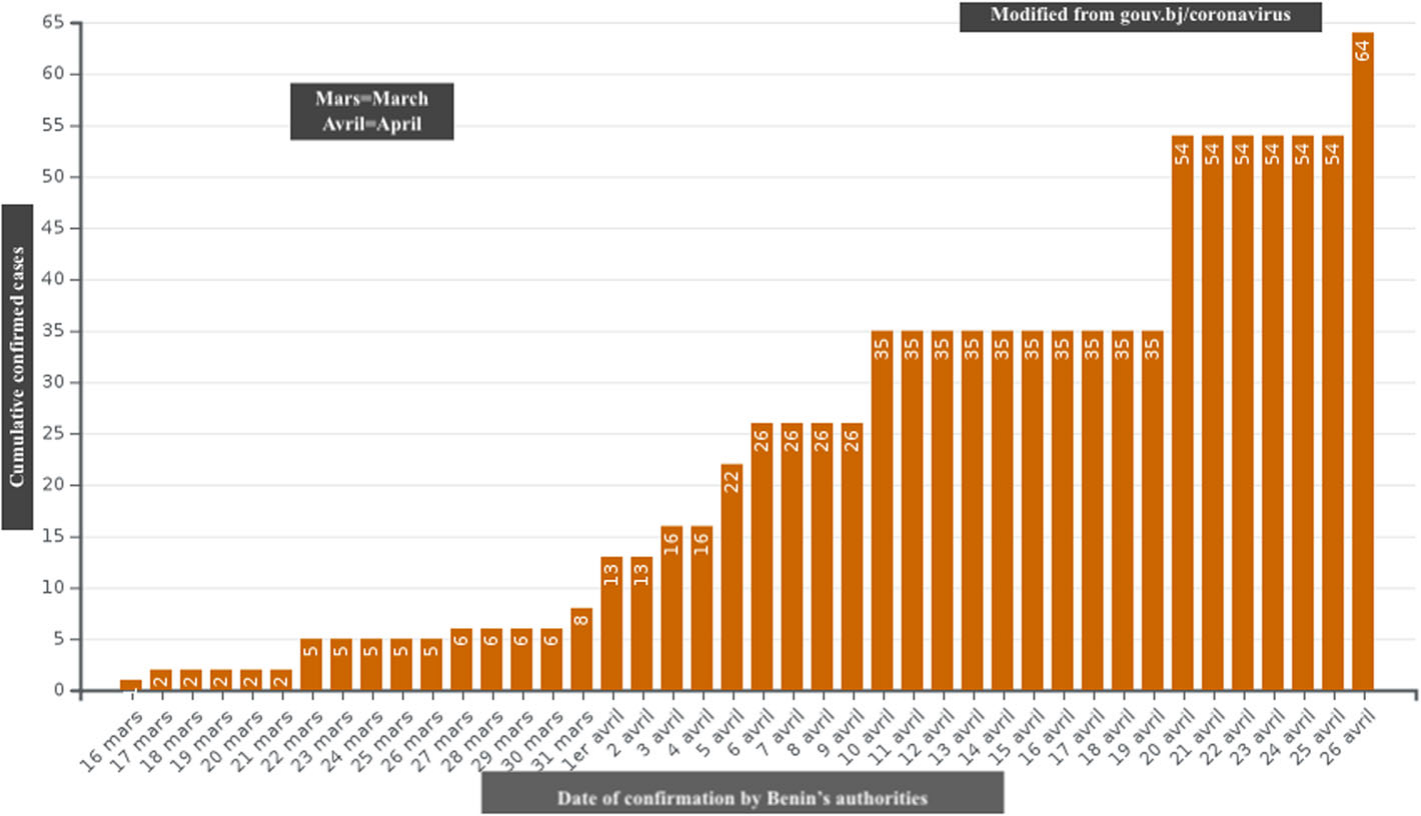
COVID-19 cases detection timeline. Data derived from “Gouvernement de la Republique du Benin” COVID-19 webpage (https://www.gouv.bj/actualites/categorie/coronavirus%2D%2Dcovid-19-/) [9]

## Conclusion

In the span of a couple of months, COVID-19 has severely challenged the world. To prevent the virus spread, nations across the world went on lockdown fully knowing the economic drawback of such measures. Conscientious of its fragile economy, Benin’s government settled on creating a sanitary cordon around the affected areas with free movement of the population within it while appealing for the public’s responsibility, promoting handwashing and hygiene along with social distancing. Although the sanitary cordon measure is effective in preserving the economic costs on the household level and containing the spread of the virus, it is however expected that people will find ways to intrude in and out of the sanitary cordon which will eventually lead to COVID-19 spreading to other towns or cities.

## Data Availability

All data used are publicly available, and sources are cited throughout.

## Abbreviations

COVID-19: Coronavirus disease 2019
WHO: World Health Organization
